# Investigating the Effects of Amino Acid Variations in Human Menin

**DOI:** 10.3390/molecules27051747

**Published:** 2022-03-07

**Authors:** Carmen Biancaniello, Antonia D’Argenio, Deborah Giordano, Serena Dotolo, Bernardina Scafuri, Anna Marabotti, Antonio d’Acierno, Roberto Tagliaferri, Angelo Facchiano

**Affiliations:** 1 Dipartimento di Scienze Aziendali, Management and Innovation Systems, Università degli Studi di Salerno, 84084 Fisciano, Italy; carmenbnl93@gmail.com (C.B.); sdotolo@unisa.it (S.D.); 2 National Research Council, Institute of Food Science, 83100 Avellino, Italy; antoniadargenio1@gmail.com (A.D.); deborah.giordano@isa.cnr.it (D.G.); dacierno.a@isa.cnr.it (A.d.); 3 Dipartimento di Chimica e Biologia “A. Zambelli”, Università degli Studi di Salerno, 84084 Fisciano, Italy; bscafuri@unisa.it (B.S.); amarabotti@unisa.it (A.M.)

**Keywords:** human menin, multiple endocrine neoplasia type 1 (MEN1), protein structure, protein function, protein modelling, missense variations

## Abstract

Human menin is a nuclear protein that participates in many cellular processes, as transcriptional regulation, DNA damage repair, cell signaling, cell division, proliferation, and migration, by interacting with many other proteins. Mutations of the gene encoding menin cause multiple endocrine neoplasia type 1 (MEN1), a rare autosomal dominant disorder associated with tumors of the endocrine glands. In order to characterize the structural and functional effects at protein level of the hundreds of missense variations, we investigated by computational methods the wild-type menin and more than 200 variants, predicting the amino acid variations that change secondary structure, solvent accessibility, salt-bridge and H-bond interactions, protein thermostability, and altering the capability to bind known protein interactors. The structural analyses are freely accessible online by means of a web interface that integrates also a 3D visualization of the structure of the wild-type and variant proteins. The results of the study offer insight into the effects of the amino acid variations in view of a more complete understanding of their pathological role.

## 1. Introduction

Menin is a tumor suppressor protein encoded by the *MEN1* gene located on chromosome 11q13 and made up of 9 introns and 10 exons. Menin is primarily a nuclear protein even if it has also been found in small amounts at the cytoplasm and membrane level. Indeed, menin contains three nuclear localization signals (NLSs) but also two nuclear exit sequences (NESs) suggesting its ability to enter and exit the nucleus [1,2,3]. This protein is highly conserved in different animal species, but it does not share sequence similarity with other known proteins, and it does not have intrinsic enzymatic activity. In order to elucidate its functions, several protein-protein interaction studies revealed that more than 50 different proteins could associate with menin. Consequently, menin was defined as a scaffold protein that interacts directly or indirectly with various partners involved in transcriptional regulation, DNA damage repair, cell signaling, cell division, proliferation, and migration [4,5]. For instance, in transcription regulation, menin has been proved to suppress gene transcription by binding the AP-1 transcription factor JunD and repressing JunD-mediated transcriptional activity [6,7], and to activate transcription binding members from the mixed-lineage leukemia (MLL) in the histone methyltransferase complexes [8]. Menin is ubiquitously expressed but it was found to have tissue-specifically opposite functions. In endocrine cells, it suppresses tumorigenesis, while in leukemogenesis it acts as a pro-oncogenic cofactor of MLL-fusion proteins derived from chromosomal rearrangements that induce acute leukemias [5,9]. The three-dimensional structure of human menin revealed more insights about its functions. X-ray diffractometry studies revealed a structure that resembles a curved left hand and consists of four single associated domains: an N-terminal domain (NTD) characterized by a long β-hairpin and essential for stability, a “thumb” domain, a “palm” domain that consists of eight alpha helices, and a C-terminal “fingers” domain. A central deep pocket is formed by the thumb and palm domains and has been shown to function as a protein-protein interaction module, as suggested by the presence of several tetratricopeptide motifs. Indeed, co-crystallization studies have shown the direct binding between this central cavity and peptides from JunD and MLL1 [10,11].

Mutations in MEN1 gene are responsible for the onset of multiple endocrine neoplasia type 1 (MEN1), a rare autosomal dominant disorder characterized by endocrine alterations that must be present in a combined manner for at least two of the following conditions: tumors of the parathyroid glands, anterior pituitary gland, and neuroendocrine tumors of the gastro-entero-pancreatic tract (GEP-NET). These conditions can be present either in a non-hereditary form with no family history of MEN1 (sporadic MEN1), or in several members of a family (familial MEN1). In addition, nonendocrine tumors such as angiofibromas, collagenomas, lipomas, and melanomas can occur. The MEN1 syndrome has variable inter- and intra-familial expression, a high degree of penetrance and, in absence of treatment, MEN1 patients have earlier mortality. Combined studies of tumor linkage and microdeletions in affected families, thanks to which the gene had been localized in 1997, revealed that MEN1 patients have usually one inherited germline mutation at MEN1 locus and somatic loss of heterozygosity or point mutation in the other allele. This supports the MEN1 role as tumor suppressor gene in endocrine tissues, in line with the Knudson hypothesis, also known as the two-hit hypothesis, according to which tumor suppressor genes require the biallelic inactivation for tumor development. In some cases, the germline mutation is not transmitted, but arises de novo and is then followed by a somatic mutation. The result of these chromosomal alterations is the loss of function of the menin protein and organ tumorigenesis [1,12,13,14].

To date, thousands of mutations, both germline and somatic, are known in the *MEN1* gene, scattered throughout its entire length with no hotspots and no phenotype-genotype correlation [15,16,17]. Most of these alterations include non-sense and frameshift mutations and, in minor fraction, also splicing defects and large deletions, causing menin inactivation due to the premature protein truncation or inhibition of its expression. About 25% of MEN1 mutations are missense variants whose consequences at the level of structure and function are not easily conceivable as for the variants mentioned above [12,18,19].

Single amino acid change induced by missense mutation may results in no relevant effect or in serious structural alterations affecting protein stability or its binding properties, thus impairing the protein function. In-depth analyses are required to discriminate between benign or pathogenic variants. The knowledge of the structural and/or functional effects of the pathogenic variations may help in finding the most appropriate therapy. Therefore, we use an in silico approach to investigate structural properties of menin and the potential impact of more than 200 missense mutations detected in patients with pathologic conditions and reported in public databases. We characterized the effects of these variations in terms of an alteration of structural features, protein thermostability and/or function of menin protein, by performing computational analyses. Finally, we stored all the results in a free-online accessible database in order to share them with the scientific community.

## 2. Results and Discussion

### 2.1. Protein Modelling of Human Menin Wild-Type and Variants

**Figure 1:**
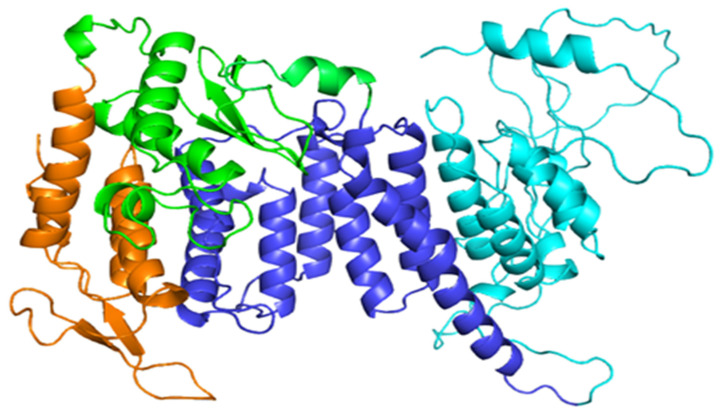
3D structure of menin model shown as cartoon colored according to the different menin domains. The N-terminal domain (NTD) is colored in orange, the thumb domain in green, the palm domain in blue, the fingers domain (C-terminal domain) in cyan.

The study of human menin crystal structures found on RCSB PDB has highlighted that all entries have different missing residues, but residues 460–536 are missing in all of them. These residues correspond to a long loop, genetically deleted because it interfered with the crystallization procedure. Therefore, in order to have a complete 3D structure of the protein to be used in this study, we created a full-chain model of human menin, selecting as templates the two X-ray structures identified by the PDB codes 3U84 and 4GQ4 [10,11]. Our model (Figure 1) offers as a benefit over the experimental structures used as a template the feature to have a complete chain that includes also the regions deleted or not visible in the experimental structures. The model showed quality improvement over the experimental structures in terms of energetic and stereochemical properties (see Supplementary Materials Tables S1 and S2). The model of the wild-type menin was then used to generate in silico the 3D models of the missense variants of this protein. The wild-type and the variants models were then analyzed for the structural properties. Data obtained were collected in the database freely available (see under Methods), from which it is also possible to download the 3D models in the PDB format. For each variant, the database offers a detailed analysis of the structural parameters in the mutated amino acid in comparison, side-by-side, to the properties of the wild-type protein. In the following paragraphs, we describe the predicted effects of variations.

### 2.2. Effects of Amino Acid Variations

Overall, the 214 variants presented in our online database indicate that the salt bridges are modified in only three cases, the secondary structure is affected in only five cases, the solvent accessibility in only 24 cases. The H-bond interactions are affected in 130 cases. The most complex effect concerns the predicted stability that is increased by amino acid variation in 18 cases, decreased in 152 cases, while predictions return uncertain effect in the remaining 44 cases. The complete summary of the effects for each variant at the level of the analyzed properties (i.e., secondary structure, solvent accessibility, stability, H-bonds, salt bridges) is reported in Supplementary Materials Table S3.

The loss or gain of interactions as H-bonds or salt bridges can result in a change of protein stability. However, there are mutations predicted to affect the protein stability without any detected effect on the parameters investigated, and they are presented in the following paragraph.

#### 2.2.1. Effects of Secondary Structure, Salt Bridges and H-Bonds

Only five amino acids variations affect the secondary structure, and they involve proline residues, i.e., P12L, H139P, T148P, L173P, L294P. The peculiar nature of proline may explain the change of secondary structure, as the backbone portion of the proline is unique and reduces the conformational space for phi-psi angles. In the case of H139, the substitution in proline changes the turn secondary structure to bend. The alpha helix conformation for the wild-type T148, L173, and L294 residue is changed by proline variation to the turn conformation. It is known that proline backbone portion may break helices, and this explains the observed effect, although in 36 other variations to proline no change in the secondary structure is observed. Similarly, the P12 substitution by leucine changes the secondary structure state, probably because the higher conformational freedom of the leucine backbone portion allows assuming a more suitable condition.

**Figure 2:**
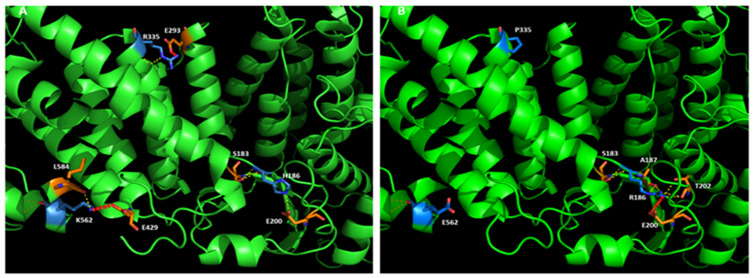
Mutations affecting salt-bridge interactions. (**A**) menin wild-type; (**B**) menin affected by three mutations (H186R, R355P, K562E). Menin backbone is represented in green with residues involved in the interactions being or not subjected to mutation are highlighted in blue and orange sticks, respectively. Salt bridges are displayed by a red dotted line, H-bonds by yellow dashed lines.

The variations that affect salt bridges are H186R, R335P, and K562E. In these cases, the effect is clearly associated with the change of the side chain charge. H186R substitution allows a salt bridge with Glu200, while the wild type residue does not make this interaction. R335P loses H-bonds with a close residue and salt bridges with Glu293 in the opposite helix. K562E, located in the second alpha/beta motif, loses an important salt bridge with Glu429 in the opposite helix (Figure 2).

The interaction affected to a larger extent by variations is the H-bond. There is loss or gain in 130 variations (details in Supplementary Materials Table S3), and in most of the cases there is an effect on the protein stability, too, as can be expected, because H-bonds contribute to the conformational stability.

#### 2.2.2. Mutations Affecting Only Protein Stability

**Figure 3:**
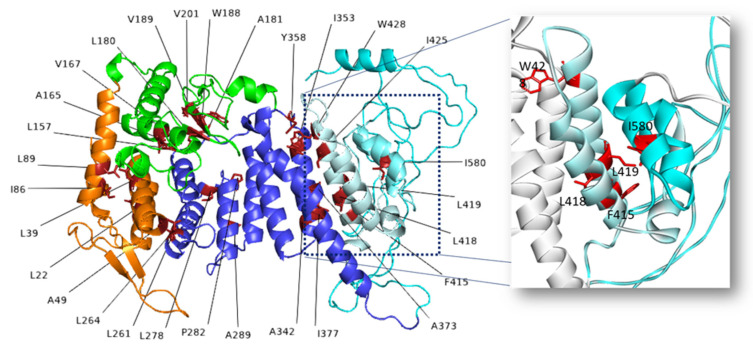
Structural position in the menin model of wild-type buried residues affected by mutations predicted as destabilizing. Affected residues are shown as red stick models: L22R, L39W, A49V, I86F, L89R, L157W, A165T, V167F, L180R, A181S, W188S, W188C, V189E, V201G, L261F, L264R, L278P, P282H, A289Q, A289E, A289V, A342D, I353N, Y358D, A373D, I377M, F415L, L418R, L419Q, I425N, W428R, and I580N. The enlarged view shows the investigated residues in the two α/β motifs colored in pale cyan and teal, respectively.

Our study has predicted that 56 amino acid mutations affect only protein stability and, among these, 47 generate fewer stable variants, whereas nine generate more stable variants. In this case, 32 out of 47 destabilizing mutations impact hydrophobic residues buried in the 3D structure (Figure 3), with substitution either by hydrophilic residues, with a possible loss of buried hydrophobic interactions, or by hydrophobic residues of larger sizes and different shapes, with a possible effect on stability due to clashes and consequent subtle structural rearrangements. A visual inspection of the position of the wild-type residues has revealed that they are located at the interface between different menin domains or between different secondary structure elements of a specific domain. In particular, the variants F415L, L418R, L419Q, and I580N are remarkable as they affect residues at the interface between two α/β motifs closely associated in the fingers’ domain of menin structure (see the enlarged view within Figure 3). This region seems to be critical, as the menin stability was greatly reduced after the deletion of the C-terminal α/β motif [10]. Moreover, these predictions are supported by experimental studies, according to which some of these mutants (L22R, I86F, A165T, V189E, and W428R) showed reduced expression levels relative to the wild-type protein, due to rapid degradation by the ubiquitin-proteosome pathway [20,21]. Therefore, these mutations are potentially deleterious for menin correct folding, making the protein more sensitive to degradation processes.

The other destabilizing mutations involve exposed or partially exposed residues in the menin structure, and the possible explanation of the predicted effect on the protein stability resides in our observation that most of the predictors detected only a modest change in protein stability for these variants. This is not surprising, given the position of mutations.

Subtle changes in predicted protein stability is also observed in most cases of uncertain prediction of the protein stability change, due to the absence of consensus among the methods that occurs in 44 variants. We investigated in detail some of these cases. Experimental data available in the literature have shown that R176W and E260K variants, found in patients with milder forms of MEN1 syndrome, were only slightly less stable than wild-type menin, and E260K retained also the biological activity on JunD-dependent transactivation [21]. The residue Arg176 is affected by another missense variant, R176Q, well known as normal polymorphism [22]. This polymorphic variant was proved as stable as the wild-type menin [21].

About the mutations predicted to be more stabilizing (G42V, R98L, S160F, N194S, P282L, P325L, P395R, G424V, S548L), the interpretation of their impact needs more investigation. Four of them (G42V, P282L, P325L, G424V) involve buried residues. G42V and G424V variants replace both a glycine with a valine. The first glycine resides in the third α-helix of NTD domain and is strictly conserved in various species, while the second is located in the first α/β motif of the fingers’ domain. No significant change was identified by our studies, in line with the highly similar properties of the replacing residues, hence the discussed alterations seem to be neutral in silico. P282L and P325L mutations affect prolines in the hydrophobic core of the palm domain, at the interface between three alpha helices. The variant P325L was experimentally found to be less stable [21].

The other alterations involve exposed residues. R98L affects a residue located at the N-terminal domain and R98 makes hydrophobic interaction with LEDGF-M418 and LEDGF-N421. The remaining variants P395R, and S548L, affect residues in unstructured and exposed regions of the fingers’ domain. For this reason, we are not able to provide an interpretation of their effect using our results, but we can hypothesize possible outcomes in protein flexibility and/or in menin-proteins binding given that the fingers domain is implicated in menin association with different proteins, such as ASK, CHESI1and Smad3, as stated in literature [23]. In the case of S548L, it is reported that S548 is a site of phosphorylation [24] and the effect of substitution can be associated with the loss of this functional aspect.

### 2.3. Effects on Protein Function

**Figure 4:**
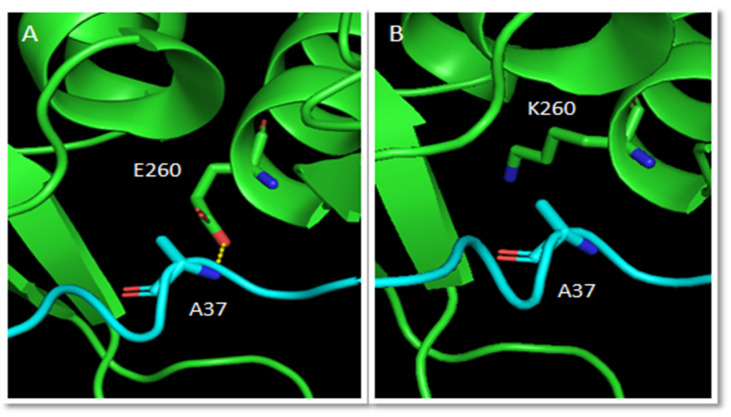
Effect of E260K variation on the interaction of menin (green) with MLL1 peptide (cyan). Labeled residues are shown in stick mode. (**A**) The H-bond between wild-type menin-E260 and MLL1-A37 is shown as a dashed yellow line. (**B**) The variation of E260 to K loses the interaction.

Menin acts as a key scaffold protein with various partners, it is possible that variations affect the interactions between menin and other proteins. As a matter of fact, 5 variations (D185A, C246F/Y, A247V, E260K), located in the menin binding pocket, replace residues directly involved in menin-proteins association, according to our computational analysis executed on the available crystal structures of human wild-type menin in complex with JUND, MLL1 and LEDGF proteins. Studying these complexes together with those built by replacing the crystal menin with the modelled one, we have verified the conservation of the interactions made by the discussed residues. More in depth, the residue Asp185 makes an electrostatic bond with MLL1-Arg12, Cys246 forms a hydrophobic interaction with MLL1-Ala11, Ala247 makes hydrophobic interactions with MLL1-Pro10 and JunD-Pro33, and Glu260 forms an H-bond with MLL1-Ala37. The analysis of the same complexes built with the menin mutants detected the loss of these contacts. An example is visible in Figure 4 showing the loss of the H-bond made by the wild-type residue Glu260 with MLL1 when replaced by a lysine.

**Figure 5:**
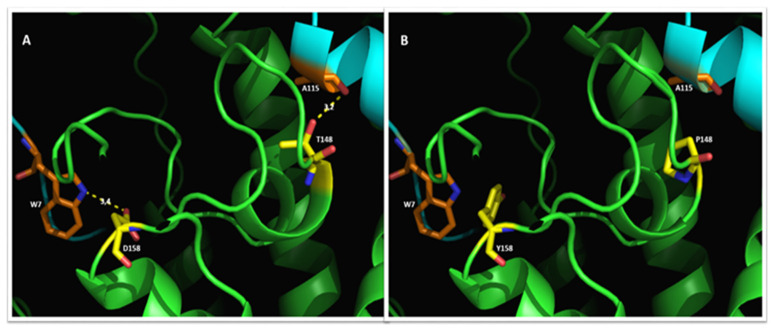
Interactions of menin (green) with MLL1 (cyan). Yellow dashed lines correspond to the H-bond. (**A**) The H-bonds between wt menin D158 and T148 (yellow sticks) with MML1 W7 and A115 (orange sticks), respectively. (**B**) Menin variations T148P and D158Y cause the loss of the two H-bonds.

Two other amino acids involved in interactions with MLL1 are also subjected to variation. T148 and D158 form H-bonds with MLL1 A115 and MLL1 W7, respectively (Figure 5), and the R148P and D158Y variations cause the loss of the H-bonds.

The mutation A247V seems to be the only exception because the valine maintains in silico the hydrophobic contacts of the original residue. In contrast, experimental studies discovered for this variant no interaction with JunD and no effect on JunD activated transcription [6] and reduced interaction with MLL1 and LEDGF [25]. Although no change in the interactions of menin with the interacting proteins was detected by our analysis, the loss of interaction may be related to the predicted instability, also found in vitro [21]. The same may occur also for the variants N57K, G110E, E116G, R137W and I147F, which concern residues flanking those directly implicated in menin-protein interactions according to our analysis. Similarly, the substitution V555L may weaken the association between the two α/β motifs, critical for menin stability as mentioned above, which is mediated by residues including the neighbour Pro554 and Phe558.

### 2.4. Amino Acid Variations without Definable Effect or with Contrasting Effects

There are 20 mutations with no predicted impact on any structural features analyzed in our study, and an uncertain effect on the protein stability. G42A is a mutation located in the NTD domain and affects a buried residue; this change seems to have no relevant effects on menin. Different is instead the case of the substitution of a glycine with arginine in position 286; despite no change in terms of intrachain interactions, the position of this substitution is in a strategic point for the binding of several interactors as MLL1 or JunD. G286 in fact is located in the menin binding pocket. This could be an explanation for the experimental studies suggesting a loss of interaction with MLL1 for menin affected by this mutation [10].

G310R and G310D are two mutations affecting a residue located in the palm domain but in a buried region, in particular in the α14 helix at the interface with α13 and α15 helices. No experimental evidence concerning structural and/or functional effects is present in literature for G310R, however, for G310D studies on mutant protein, expression levels state that this mutation is strongly destabilizing for menin protein in patient affected by familial isolated hyperparathyroidism [21]. Our results do not indicate changes in the intra-chain interactions; however, predictors about stability are strongly in conflict predicting for half a strongly destabilizing effect and the other half a stabilizing effect. All three helices show the particular feature to have a glycine residue in the middle, and α13 and α15 helices form part of the main menin binding pocket. Probably the mutation of one of those residues could have an impact on the structural stability due to still unknown functions fulfilled by α14 helix.

**Figure 6:**
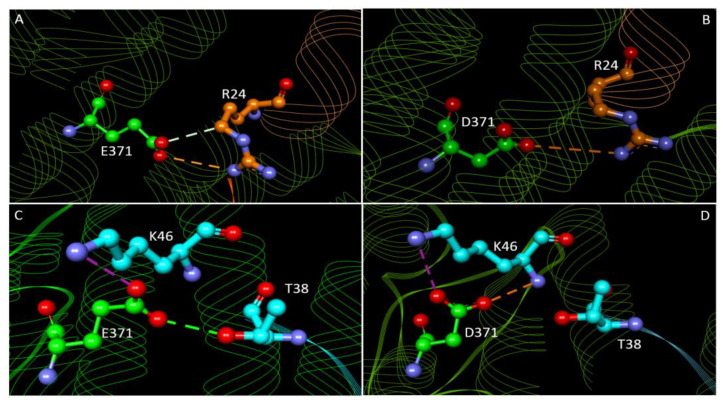
Detail of menin-MLL1 and menin-JunD interactions related to residue E371 and its mutation. (**A**) interactions between menin wild type E371 and MLL1 R24; (**B**) interaction between menin E371D variant and MLL1. (**C**) interactions between menin wild type and JunD; (**D**) interactions between menin mutated in E371D and JunD. Menin is represented in green cartons with residues E/D371 involved in the interactions highlighted in green balls and sticks. MLL1 and JunD are represented in orange and cyan cartons, respectively, with residues involved in the interactions highlighted in orange/cyan balls and sticks. Green dashed lines correspond to the H-bond, violet dashed lines to salt-bridges, orange dashed lines to electrostatic interaction, white dashed line to Carbon H-bond.

About the mutation E371D, experimental studies have shown no effect on protein stability [21]; moreover, this variant is present in population databases (rs149383809, Exome Aggregation Consortium ExAC 0.01%) and has an allele count higher than expected for a pathogenic variant. Our results support the non-pathogenic nature of this variant, as no structural feature is affected by Glu substitution by Asp. However, the variant results less stable for 3 out 5 of the predictors. Moreover, E371 is involved in the interaction with MLL1 and JunD making in the first case a carbon H-bond and an electrostatic bond with MLL1-R24 and in the second case an H-bond with T38 and a salt bridge with K46 of the interactor. The aspartate substitution results in the loss of the H-bonds, but it could preserve the other electrostatic interactions with the positive charge of K46, although at a different distance due to the shorter side chain (Figure 6). These results are consistent with the similar properties of the wild type and mutated residues.

## 3. Methods

Different transcripts variants of *MEN1* gene exist due to alternative splicing events. Alternative splicing site at the end of exon 2 produces two menin isoforms of 615- and 610-amino acid length, which differ for five additional amino acids after codon 148, as reported in sequence databases (as an example, see the Uniprot entry: https://www.uniprot.org/uniprot/O00255, accessed on 8 February 2022). In most publications, menin mutations are annotated according to the isoform 2 (610 amino acids), while in different databases their position is numbered in relation to the 615 version. The present study is focused on the protein isoform 1 (615 amino acids) which is the proposed standard reference for menin, and the position of the investigated mutations is reported accordingly. The experimental structures of human menin protein have been searched on the online database RCSB PDB [26]. The 37 crystal structures available were analyzed comparing resolution, R-value, R-free, Ramachandran plot, z-score, missing atoms, missing residues, eventual mutations and/or deletions in order to find the best ones for the following processing [27]. These analyses were performed examining the pdb files of each structure and using the web servers Procheck [28] and Prosa-web [29] for Ramachandran plot and z-score evaluation, respectively. The structures identified by the PDB IDs 3U84 and 4GQ4 [10,11] have better parameters (Supplementary Materials Table S1) and were used as templates in order to build a model of the canonical sequence of human menin, corresponding to the 615-amino acid isoform reported in Uniprot (identifier: O00255-1). For this purpose, we used the program MODELLER 9.22 [30]. We modelled 10 models that were compared in terms of Ramachandran plot, z-score, QMEAN [31] and RMSD, with the latter two parameters computed using the webserver Swiss model [32] and the software Pymol (https://pymol.org/2, accessed on 8 February 2022), respectively. Supplementary Materials Table S2 shows that all the models obtained have good and similar parameters, but model no. 8 has the lowest z-score (−10.09) and the higher Qmean value (0.66); for these reasons model no. 8 was chosen as the reference structure for further analyses.

We used this structure to model missense mutations of menin protein extracted from four databases: Uniprot (https://www.uniprot.org, accessed on 8 February 2022) [33], ClinVaR (https://www.ncbi.nlm.nih.gov/clinvar, accessed on 8 February 2022) [34], UMD-MEN1 database (http://www.umd.be/MEN1, accessed on 8 February 2022) [35], and HGMD (http://www.hgmd.cf.ac.uk/ac/index.php, accessed on 8 February 2022) [36]. In our study, we selected only mutations reported in the literature and found in patients having pathologies, for a total amount of 219 variants. In all databases investigated, the position of mutations refers to the 610-residue isoform (Uniprot code: O00255-2). In the present work, the selected 219 mutations have been re-annotated according to the 615-residue isoform. Their modelling was made using the python script Mutate_model [37], the program MODELLER 9.22, and the pdb file of the model built before as input. Then, we predicted the impact of these variants on menin structure and function using an automated procedure to execute different programs, as already carried out in previous and similar works on galactosemia-related proteins [38,39]. In detail, the wild-type structure and the mutant models were analyzed with the programs DSSP [40] to identify the secondary structures, HBPLUS [41] to find H-bonds, NACCESS [42] to calculate the solvent accessibility of each residue and an in house script to determine the presence of salt bridges between positive and negative residues. Each variation in these features between the wild-type and the mutant proteins was detected. In addition, we predicted the effect of these mutations on the protein stability by means of five different web servers: MAESTROweb [43], INPS-3D [44], PoPMuSiC [45], DynaMut [46], and DUET [47]. The selection of these tools is because they use different approaches [48,49]. In each predictor, we used the wild-type model as the starting point to make the calculations. The final result derives from the consensus of these servers: the mutation was classified as “more stable” or “less stable” when at least 3 out of 5 predictors gave the same result, while it was classified as “uncertain” when the consensus of the predictors was not achieved. The results of all the analyses made on wild-type and mutant models were stored in a freely accessible database (http://bioinformatica.isa.cnr.it/menin-protein-db, accessed on 8 February 2022 or http://www.protein-variants.eu/menin-protein-db, accessed on 8 February 2022). The database structure is based on the same architecture developed by our previous studies [38,39,50,51]. For each variant, the database offers a page with details concerning the analyzed structural properties, with a side-to-side comparison to the properties in the wild-type protein. Structural properties reported are the secondary structure and the phi and psi angles, the solvent accessibility, the intra-chain interactions in terms of H-bonds, salt bridges, hydrophobic interactions, in all cases with details of the atom linked. Relative solvent accessibility is reported (i.e., the percentage of solvent exposed surface), and residues are considered buried when they have ≤9% of accessible surface area, exposed when they have >36% of accessible surface area, and partially exposed in the middle range. Moreover, the page reports the prediction of the effect of variation on protein stability The online database includes 214 out of the 219 variants, and the analyses reported under Results and Discussion refer to the variants in the database online. Finally, given the existence of menin crystal structures in complex with some interacting proteins, we investigated them with the PDBePISA tool [52] and Discovery Studio Visualizer software (https://www.3ds.com, accessed on 8 February 2022) in order to identify residues required for protein-protein interactions. The investigated structures are identified by PDB IDs 3U85 (menin in complex with MLL1 peptide with sequence S_5_RWRFPARP_13_), 3U86 (menin in complex with JunD peptide with sequence R_30_LFPGAPTA_39_K_46_K_47_), 3U88 (menin in complex with MLL1 and LEDGF peptides of 75 and 89 residues, respectively), and 4GQ6 (menin in complex with MLL peptide with sequence S_4_ARWRFPARPGT_15_). Moreover, to investigate the effects of variations on the interaction with MLL1, JunD, and LEDGF, we generated models of the menin variants in complex with the interactors. The resulting variant complexes were analyzed in the same way and compared with the wild-type-complex counterpart to find out any changes in intermolecular bonds.

## 4. Conclusions

The work presented is a comprehensive examination of the known amino acid mutations affecting menin, with relation to different pathological conditions. Menin mutations are not clusterized in specific domains but are spread along the whole sequence, as expected considering the multiple functions of menin as a scaffold protein. In order to elucidate a potential pathogenic role of these variants, their effects at a structural and functional level have been investigated and described using a computational approach and in comparison to experimental evidence reported in the literature.

According to the collected results, it was possible to detect three main different groups of variants: mutations with multiple effects (on stability, structure and intra-chain interactions), mutations affecting only thermodynamic stability, and mutations with apparently not definable effect.

The majority of the analyzed mutations falls in the first group, with changes at two or more observable features (i.e., secondary structures, solvent accessibility, loss or gain of interactions as H-bond and/or salt-bridge formations). Therefore, variations of this group are probably able to alter protein integrity and function.

About the second group, mutations replacing buried residues have a more destabilizing effect due to the critical location at the interface between different menin domains. This is confirmed by experimental evidence showing a reduction of expression levels for some of these variants owing to the rapid proteasome degradation process. Differently, variants replacing exposed residues have a minor destabilizing effect on menin integrity. Few mutations have been identified as more stabilizing.

Lastly, 20 mutations have been reported to have a not definable effect, thus they could be harmless for menin correctly folding.

Analysis performed on mutated and wild-type menin-interactor complexes have highlighted that mutations classified in different groups could impair menin interaction with the investigated protein partners. This computational evidence regards predominantly residues located in the well-known menin binding pocket, whose interactions with the menin partners are well characterized by structural complexes available in PDB. However, in view of the scaffold nature of this protein, it is reasonable to hypothesize that also variants affecting exposed residues can explicate a similar effect with other binding proteins for which no structural data are available to date, but whose presence is demonstrated by literature.

On the whole, our results are in good agreement with experimental studies performed on the most known mutations, confirming the validity of the implemented approach and the confidence of the obtained results. It is in our planning the periodical update of the database to add novel variations discovered and reported in the literature. Further enhancements of the methodology applied to investigate the structural and functional effects of amino acid substitutions are also planned, as the addition of more structural analyses and predictions on the effects of mutations. As an example, amino acid substitution may increase the propensity to amyloid formation in proteins, as well recognized in the literature [53,54,55]. Predictive methods have been developed [56] so that they can be considered to extend the predictions in the framework of our approach. Although in the case of menin there is no evidence in the literature of a possible amyloid formation, this is an interesting topic to be taken into consideration when all possible effects of mutations are under investigation by predictive tools. The detailed knowledge of these effects may be useful to design appropriate drugs or define therapies focused on the effect to be counterbalanced. For instance, pharmacological molecules designed to restore protein stability could be used for the treatment of patients affected by menin mutations impairing only this feature.

In summary, this work allows to decode the impact of several missense variants on menin structure and function performing in-depth in silico analysis. Results are freely accessible online for the development of more advantageous therapeutic strategies oriented to personalized medicine.

## Data Availability

Data are available as Supplementary Materials and at the online database web site (http://bioinformatica.isa.cnr.it/menin-protein-db, accessed on 8 February 2022 or http://www.protein-variants.eu, accessed on 8 February 2022 or http://www.protein-variants.eu/menin-protein-db, accessed on 8 February 2022).

## Supplementary Materials

Table S1: Stereochemical and structural features of the experimental structures of human menin, identified by PDB IDs 3U83 and 4GQ4, Table S2: Analysis of the 10 models of human menin obtained with Modeller in terms of Z-score, QMEAN, RMSD with templates, and Ramachandran plot parameters, Table S3: Summary of the effects of amino acid variations, Table S4: Results of predictions of the effects of amino acid variations on protein stability.
