# Supplementary material for: Investigating the Effects of Amino Acid Variations in Human Menin
Source: Molecules. 2022 Mar 7;27(5):1747. doi: 10.3390/molecules27051747 (PMC8911756; doi:10.3390/molecules27051747)
Supplement: Supplementary file 1 [file molecules-27-01747-s001.zip › Supplementary-Table-S4.pdf]

Supplementary Table S4. Predictions of the stability effect of mutations

Results from the predictions are reported, with the color code: red = less stable, blue = more stable, black = uncertain effect.

Consensus column reports the final prediction, used in the online database, with the same color code.

MENIN							
MISSENSE MUTATION	Position	Dynamut1	INPS 3D (ΔΔG) kcal/mol	DUET- Predicted Stability Change (ΔΔG)	MAESTROweb (ΔΔG)	Popmusic	CONSENSUS
P12L	12	0.093	-0.470	0.142	-0.247	1.35	3/5
L22P	22	-1.321	-3.491	-1.888	0.357	3.74	5/5
L22R	22	-2.087	-1.980	-2.210	0.038	2.02	5/5
E26K	26	0.053	-0.469	-1.186	0.076	1.04	3/5
L27P	27	-1.620	-3.465	-2.298	0.329	3.24	5/5
L37P	37	-1.729	-3.525	-2.115	0.700	3.61	5/5
S38F	38	-0.480	-0.038	-0.576	-0.383	0.22	3/5
S38P	38	-0.451	-1.068	-0.207	0.000	2.13	4/5
L39W	39	-1.089	-2.088	-2.073	-0.121	1.51	4/5
G42A	42	1.251	-0.978	-0.212	-0.215	0.17	uncertain
G42D	42	1.086	-0.998	-2.306	-0.151	0.84	uncertain
G42S	42	1.547	-1.081	-1.726	-0.241	0.82	3/5
G42V	42	1.731	-0.769	0.409	-0.337	1.07	3/5
E45A	45	-1.906	-0.252	-2.666	-0.017	1.17	3/5
E45D	45	-1.379	-0.813	-2.010	-0.065	1.2	3/5
E45G	45	-2.671	-1.095	-3.448	0.305	1.5	5/5
E45K	45	-1.965	-0.374	-2.834	0.090	1.09	4/5
E45Q	45	-1.209	-0.352	-2.503	-0.107	0.17	3/5
E45V	45	-1.067	0.917	-1.945	-0.085	0.18	3/5
A49V	49	0.012	-0.764	-0.529	0.045	0.74	3/5

R52G	52	-0.478	-1.282	-0.438	0.117	0.64	5/5
V53I	53	-0.164	0.120	0.064	-0.126	0.23	uncertain
N57K	57	-0.057	-0.014	0.449	0.016	0.08	3/5
P72H	72	0.593	0.197	0.261	0.144	0	uncertain
I86F	86	-1.095	-1.342	-1.445	-0.071	1.5	4/5
L89R	89	-0.657	-2.109	-1.687	0.060	1.27	5/5
R98L	98	-0.158	0.081	0.439	-0.434	-0.27	3/5
G110E	110	-1.177	0.233	-1.296	-0.315	0.15	3/5
E116G	116	-1.174	-0.949	-1.148	0.035	1.07	4/5
W126G	126	-1.605	-2.260	-2.518	0.231	3.27	5/5
K135I	135	0.831	0.343	0.214	0.021	0.22	uncertain
R137W	137	0.112	-0.858	-0.302	-0.027	0.04	uncertain
H139D	139	-0.555	-1.117	-0.610	0.171	1.61	5/5
H139N	139	-0.632	-0.910	-0.665	0.151	0.73	4/5
H139P	139	-0.946	-1.021	-0.510	0.117	1.87	5/5
H139R	139	0.100	-0.671	-1.046	0.057	1.34	3/5
H139Y	139	0.673	-0.206	0.398	-0.087	0.81	3/5
Q141R	141	0.545	-0.843	0.197	0.051	0.5	uncertain
F144C	144	-1.085	-2.610	-1.631	-0.276	2	4/5
F144V	144	-0.390	-2.288	-1.302	-0.188	1.79	4/5
S145R	145	1.155	-0.698	-0.953	-0.292	0.78	uncertain
F146S	146	-2.484	-2.881	-3.060	0.434	3.47	5/5
I147F	147	0.421	-0.843	-1.297	-0.132	0.97	uncertain
T148P	148	-0.371	-1.258	-1.336	0.095	1.75	5/5
L157W	157	-0.226	-2.400	-1.950	0.147	0.96	5/5
D158E	158	0.445	-0.490	-0.770	0.007	1.27	3/5
D158V	158	0.449	0.890	0.289	-0.143	0.84	3/5
D158Y	158	0.409	0.368	-0.297	-0.123	0.44	uncertain
S159I	159	0.347	-0.574	0.647	-0.402	0.57	3/5
S160F	160	0.813	1.533	-0.728	-0.474	0.52	3/5
G161C	161	1.225	-1.232	-0.918	-0.246	0.08	3/5

G161D	161	0.340	-1.029	-1.210	0.042	0.91	4/5
G161R	161	0.188	-0.787	-0.828	-0.174	0.42	uncertain
G161S	161	0.835	-0.828	-1.297	-0.050	0.69	uncertain
G161V	161	1.624	-1.189	0.503	-0.260	-0.14	4/5
A163D	163	-1.659	-2.216	-2.192	0.111	1.88	5/5
F164C	164	-1.583	-2.566	-1.852	-0.004	1.87	4/5
A165P	165	-0.373	-2.000	0.374	0.142	3.26	4/5
A165T	165	-0.585	-0.877	-0.375	0.001	1.63	4/5
V167F	167	-0.153	-1.986	-1.646	-0.199	1	4/5
G168R	168	-0.353	-0.229	-1.032	-0.464	0.23	3/5
A169D	169	-1.134	-2.112	-1.830	0.114	3.12	5/5
C170R	170	-1.165	-2.059	-0.676	0.046	1.81	5/5
C170Y	170	-0.624	-2.015	-1.349	-0.288	0.66	4/5
L173P	173	-1.139	-3.249	-1.714	0.270	4.13	5/5
R176Q	176	-0.151	-0.479	-0.211	0.012	0.12	5/5
R176W	176	0.107	-0.492	-0.211	-0.060	0.39	uncertain
D177V	177	-0.393	0.388	0.213	-0.244	0.82	uncertain
D177Y	177	0.832	0.040	-0.290	-0.099	0.41	uncertain
L180R	180	-0.873	-1.923	-2.176	0.284	2.93	5/5
A181P	181	-0.001	-1.874	-0.611	-0.055	2.13	4/5
A181S	181	-1.064	-1.044	-1.765	0.047	0.83	5/5
L182P	182	-0.656	-3.218	-1.644	0.354	3.06	5/5
E184D	184	-1.248	-1.165	-1.502	0.164	0.68	5/5
E184K	184	0.212	-0.914	0.044	0.044	0.63	uncertain
E184Q	184	-0.196	-0.815	-0.108	-0.085	0.57	3/5
D185A	185	-0.219	-0.448	-0.005	-0.108	1.04	3/5
H186D	186	-1.195	-0.969	-1.905	-0.142	2.83	3/5
H186R	186	0.251	-0.260	-1.078	-0.077	1.77	uncertain
W188C	188	0.579	-1.999	-1.584	-0.223	2.84	3/5
W188R	188	0.120	-1.935	-2.092	0.127	3.32	4/5
W188S	188	-0.124	-2.905	-3.281	0.224	4.34	5/5

V189E	189	-2.454	-2.586	-3.201	0.664	5.03	5/5
P193L	193	0.804	-0.576	0.173	0.030	0.7	uncertain
N194S	194	0.035	-0.078	0.171	-0.029	0.2	3/5
Q197K	197	-0.498	-0.275	0.207	-0.228	0.36	uncertain
T198I	198	1.396	-0.879	0.494	-0.307	0.63	3/5
E200G	200	-2.379	-1.501	-2.448	-0.455	1.69	4/5
V201G	201	-1.886	-3.863	-2.709	0.385	4.11	5/5
T202I	202	0.515	-1.222	0.499	0.007	0.55	3/5
H204D	204	-0.339	-1.457	-0.267	0.268	2.6	5/5
V220F	220	1.352	-1.057	-1.030	-0.334	0.47	3/5
V220M	220	1.018	-0.585	-0.439	-0.334	0.42	uncertain
W225L	225	-1.442	-1.893	-2.342	-0.063	2.43	4/5
W225R	225	-0.550	-2.118	-2.009	0.218	3.03	5/5
W225S	225	-2.883	-2.906	-3.035	0.191	4.31	5/5
L228P	228	-1.734	-2.876	-2.633	0.541	3.32	5/5
G230R	230	0.225	-0.597	-0.838	-0.063	0.91	uncertain
S231P	231	-0.024	-1.010	-0.174	0.021	2.59	5/5
R234H	234	-0.772	-0.284	-1.305	0.038	0.1	4/5
R234L	234	-0.679	0.446	0.413	-0.151	-0.11	uncertain
D236H	236	0.653	-0.229	-0.734	0.058	0.48	3/5
E240K	240	-1.482	-0.661	-2.236	0.163	0.75	4/5
V245F	245	-0.761	-2.393	-1.424	-0.214	1.11	4/5
C246F	246	1.205	-1.811	-0.911	-0.277	0.69	3/5
C246R	246	0.063	-1.832	-0.544	-0.132	0	uncertain
C246Y	246	1.266	-1.643	-0.944	-0.326	0.41	3/5
A247V	247	0.741	-1.034	-0.282	-0.279	1.04	3/5
I252T	252	-0.420	-2.064	-0.649	-0.074	0.98	4/5
L254P	254	-0.431	-0.791	-0.067	-0.067	-1.1	uncertain
S258L	258	0.667	-0.476	-0.010	-0.208	0.91	uncertain
S258P	258	0.623	-1.422	-0.439	0.092	0.61	4/5
S258W	258	0.635	-0.944	-1.185	-0.134	0.85	uncertain

E260K	260	0.692	-0.959	0.314	-0.026	0.84	uncertain
L261F	261	-1.010	-1.250	-1.804	-0.072	0.55	4/5
Q263H	263	0.888	-0.368	-0.744	-0.357	0.64	uncertain
L264P	264	-1.684	-3.611	-2.125	0.503	4.32	5/5
L264R	264	-1.912	-2.045	-2.249	0.049	2.02	5/5
Q265P	265	-1.353	-1.234	-1.049	0.193	1.99	5/5
Q265R	265	-0.070	-0.741	-0.935	-0.051	0.78	3/5
L269P	269	-2.134	-3.509	-2.459	0.646	4.37	5/5
L272P	272	-1.605	-3.531	-2.427	0.457	4.22	5/5
L278P	278	-0.804	-3.164	-1.572	0.176	3.02	5/5
E279A	279	-0.082	-0.127	-0.540	-0.102	0.39	3/5
R280K	280	0.329	-0.296	-0.766	0.080	0.62	3/5
P282H	282	-0.504	-0.626	-1.750	-0.236	1.64	3/5
P282L	282	1.587	-0.515	0.716	-0.345	1.07	3/5
G286R	286	0.473	-0.517	-1.125	-0.475	0.02	uncertain
A289E	289	-1.145	-1.620	-2.864	-0.024	2.44	4/5
A289P	289	-0.600	-1.620	-1.340	0.040	3.03	5/5
A289Q	289	-1.399	-1.405	-1.896	-0.134	1.77	4/5
A289V	289	-1.239	-1.557	-0.541	-0.238	0.65	4/5
L291P	291	-1.218	-3.224	-2.165	0.077	3.07	5/5
L294P	294	-0.300	-2.048	-0.273	-0.392	0.79	4/5
G310D	310	0.473	-0.896	-2.323	-0.135	1.3	uncertain
G310R	310	0.030	-0.638	-1.083	-0.188	0.58	uncertain
A314P	314	-1.050	-1.725	-1.387	-0.103	1.36	4/5
T316P	316	0.586	-0.743	-0.375	-0.015	0.42	uncertain
R319P	319	-0.853	-0.918	-0.491	-0.073	1.46	3/5
D320Y	320	1.177	-0.134	0.308	-0.137	0.06	3/5
H322R	322	-0.386	-0.708	-1.954	0.137	1.68	4/5
H322Y	322	0.043	-0.281	-0.187	0.048	0.65	3/5
P325L	325	1.972	-0.364	0.156	-0.511	-0.14	4/5
P325R	325	0.632	-0.269	-1.635	-0.110	0.34	uncertain

A330P	330	-1.141	-1.681	-0.987	0.058	2.99	5/5
R335P	335	-0.631	-0.965	-0.803	-0.239	1.21	3/5
A342D	342	-0.328	-2.042	-3.154	0.010	2.12	5/5
A342P	342	-0.970	-2.094	-1.082	-0.011	2.74	4/5
L343P	343	-1.606	-3.465	-2.210	0.369	3.98	5/5
A345T	345	-0.138	-0.811	-1.687	-0.101	0.85	3/5
W346R	346	-1.239	-2.067	-1.917	0.304	2.92	5/5
A347P	347	-1.240	-1.847	-1.295	0.059	2.31	5/5
T349R	349	-0.969	-0.859	-0.624	-0.143	0.36	3/5
I353N	353	-2.347	-3.274	-2.873	0.384	3.35	5/5
D355V	355	-0.092	0.012	0.761	-0.358	0.69	uncertain
Y356N	356	-1.949	-1.832	-3.434	0.521	3.58	5/5
Y358D	358	-2.741	-2.417	-3.286	0.354	3.02	5/5
C359F	359	0.615	-1.461	-1.241	0.013	1.41	4/5
R360W	360	0.092	-0.928	-0.165	-0.343	0.05	uncertain
D362H	362	0.300	0.172	-0.173	0.024	0.2	3/5
E364K	364	0.292	-0.294	-0.391	-0.146	0.3	uncertain
F369C	369	-0.569	-2.873	-1.028	0.045	1.52	5/5
E371D	371	-0.925	-0.203	-1.108	-0.374	0.47	3/5
A373D	373	-0.060	-2.006	-2.051	0.270	2.68	5/5
A373P	373	0.146	-1.943	-0.574	0.295	2.99	4/5
I377M	377	0.185	-1.152	-0.530	-0.185	1.01	3/5
P378A	378	-1.312	-0.981	-1.285	-0.228	0.82	3/5
P378L	378	0.298	-0.523	-0.021	-0.448	-0.07	3/5
P378S	378	-0.793	-1.288	-2.239	-0.170	0.34	4/5
L380P	380	-2.272	-3.071	-2.234	0.392	4.27	5/5
L381P	381	-1.367	-3.206	-1.973	0.307	3.75	5/5
A390V	390	0.235	0.455	-0.231	-0.226	0.05	uncertain
P395R	395	0.065	0.187	0.177	-0.107	0.19	3/5
F415L	415	-0.498	-1.744	-0.908	-0.159	1.01	4/5
A416P	416	0.546	-1.797	-0.836	-0.071	1.77	3/5

L418P	418	-0.761	-3.446	-1.640	0.635	3.62	5/5
L418R	418	-2.053	-2.030	-2.015	0.186	1.85	5/5
L419P	419	-0.830	-3.518	-1.480	0.665	3.96	5/5
L419Q	419	-2.186	-2.552	-1.983	0.193	2.38	5/5
R420P	420	-0.456	-1.258	-1.006	-0.148	1.72	4/5
D423H	423	-0.744	-0.136	-2.210	-0.006	0.19	3/5
D423N	423	-1.210	-0.409	-1.898	-0.016	0.66	3/5
G424V	424	2.135	-0.953	-0.124	-0.271	-0.73	3/5
I425N	425	-1.116	-3.119	-2.709	0.409	4.28	5/5
C426Y	426	-0.315	-2.048	-1.524	-0.272	0.56	4/5
W428R	428	-0.555	-1.810	-1.810	0.267	2.82	5/5
W428S	428	-1.599	-2.799	-2.578	0.419	3.51	5/5
S432I	432	-0.333	-1.004	0.310	-0.148	0.83	3/5
S432R	432	0.406	-0.402	-0.943	0.015	0.75	3/5
T434K	434	0.046	-0.584	-0.334	-0.139	1.22	uncertain
W441C	441	-1.027	-2.097	-1.223	0.061	2.76	5/5
W441R	441	-1.552	-2.213	-2.133	0.258	2.31	5/5
W441S	441	-2.137	-3.118	-2.907	0.324	3.62	5/5
S448Y	448	0.481	0.093	-0.181	-0.375	0.51	uncertain
L449P	449	-0.712	-3.382	-1.213	0.413	3.79	5/5
F452L	452	-0.626	-2.512	-0.888	-0.148	1.78	4/5
F452S	452	-2.976	-3.261	-2.958	0.382	3.72	5/5
R457W	457	1.084	-0.277	-0.891	-0.169	0.33	uncertain
W476C	476	-0.047	-0.576	0.456	0.785	-0.27	uncertain
K507M	507	0.098	0.097	-0.116	0.609	-0.16	uncertain
G508D	508	0.755	-0.010	-0.379	-0.215	0.4	uncertain
Q513K	513	-0.156	-0.049	0.139	0.356	-0.18	uncertain
R521G	521	0.027	-1.091	-0.027	0.126	0.94	4/5
G537C	537	-0.668	0.034	-0.870	-0.201	-0.41	uncertain
A540V	540	0.580	0.100	-0.379	0.082	-0.11	uncertain
P545S	545	-0.089	0.005	-0.224	-0.032	0.47	3/5

T546A	546	-0.567	-0.071	0.270	-0.077	-0.06	uncertain
S548L	548	0.771	-0.219	0.571	-0.083	-0.01	4/5
P549S	549	-0.298	-0.157	-0.204	-0.042	0.51	3/5
V555L	555	0.112	-0.507	-0.345	-0.149	0.83	uncertain
T557S	557	-0.529	-0.871	-1.059	0.045	1.24	4/5
S560N	560	-0.189	-0.438	-1.229	0.190	1.45	4/5
S560R	560	-0.566	-0.259	-0.757	0.117	2.32	4/5
K562E	562	-0.931	-1.292	-1.185	-0.402	0.51	4/5
M563K	563	-1.167	-1.763	-1.219	0.186	2.37	5/5
M566K	566	-1.233	-1.812	-1.300	0.162	2.73	5/5
M566R	566	-0.873	-1.401	-1.201	0.042	2.49	5/5
M566T	566	-0.988	-2.018	-2.169	0.097	2.22	5/5
I580N	580	-2.079	-3.198	-2.848	0.209	3.16	5/5
L584P	584	-1.202	-3.363	-2.321	0.314	2.44	5/5
S588P	588	-0.274	-0.721	-0.139	-0.131	1.19	3/5
