# Supplementary material for: Investigating the Effects of Amino Acid Variations in Human Menin
Source: Molecules. 2022 Mar 7;27(5):1747. doi: 10.3390/molecules27051747 (PMC8911756; doi:10.3390/molecules27051747)
Supplement: Supplementary file 1 [file molecules-27-01747-s001.zip › Supplementary-Table-S1.pdf]

Supplementary Table S1

Stereochemical and structural features of the experimental structures of human menin, identified by PDB IDs 3U83 and 4GQ4.

FEATURES	3U84	4GQ4
**RESOLUTION (Å)**	2.50	1.27
**R VALUE**	0.198	0.149
**R FREE**	0.236	0.182
**RAMACHANDRAN PLOT**	88.8% core, 11.0% allow., 0.1% gener., 0.0% disall	93.3% core, 6.2% allow., 0.5% gener., 0.0% disall.
**Z-SCORE**	-9.78 [chain A (506 aa)]	-9.62 [chain A (468 aa)]
**MISSING RESIDUES**	YES	YES
**DELETION**	YES (460-536)	YES (54-73; 387-398; 460-536)
