# Supplementary material for: Investigating the Effects of Amino Acid Variations in Human Menin
Source: Molecules. 2022 Mar 7;27(5):1747. doi: 10.3390/molecules27051747 (PMC8911756; doi:10.3390/molecules27051747)
Supplement: Supplementary file 1 [file molecules-27-01747-s001.zip › Supplementary-Table-S2.pdf]

Supplementary Table S2

Analysis of the ten models of human menin obtained with Modeller, in terms of Z-score, QMEAN, RMSD with templates, and Ramachandran plot parameters.

Models	PROSAweb Z-score	QMEANDisCo	RMSD (3U84)	RMSD (4GQ4)	RAMACHANDRAN PLOT ANALYSIS (%) core	allowed	generously allowed	disallowed
Model 1	-9.64	0.64	0.427	0.368	92.9	6.0	0.8	0.4 (Val153;Gln405)
Model 2	-9.51	0.61	0.637	0.378	89.8	7.7	1.7	0.8 (Leu13; Asn379; Ala528; Gln589)
Model 3	-9.76	0.62	0.640	0.625	90.0	8.7	1.0	0.4 (Ser151; Glu482)
Model 4	-9.72	0.64	0.547	0.685	91.1	7.3	0.4	1.2 (Phe134; Glu209; Gln298; Gln403; Gln405; Glu535)
Model 5	-9.51	0.65	0.496	0.254	93.1	6.0	0.4	0.6 (Glu479; Arg485;Arg532)
Model 6	-9.84	0.63	0.602	0.338	89.0	9.3	1.4	0.4 (Gln473; Glu552)
Model 7	-9.97	0.64	0.556	0.306	91.5	6.2	1.4	1.0 (Phe134; Asn194; Val527; Gln589; Lys596)
Model 8	-10.09	0.66	0.563	0.531	91.9	5.9	0.6	0.6 (Ala68; Ser538; Gln589)
Model 9	-9.71	0.63	0.485	0.375	89.8	8.7	1.0	0.6 (Glu209; Glu491; Gln589)
Model 10	-9.39	0.65	0.629	0.295	91.1	7.1	1.0	0.8 (Lys135; Glu209; Val527; Gln589)
