# Supplementary material for: Investigating the Effects of Amino Acid Variations in Human Menin
Source: Molecules. 2022 Mar 7;27(5):1747. doi: 10.3390/molecules27051747 (PMC8911756; doi:10.3390/molecules27051747)
Supplement: Supplementary file 1 [file molecules-27-01747-s001.zip › Supplementary-Table-S3.pdf]

Supplementary Table S3. Summary of the effects

Residue	Exon	Codon	Amino Acid	Secondary Structure	Solvent Accessibility	Predicted Stability	Intrachain Interactions	H-bonds	Salt Bridges
12	1	CCG→CTG	PRO→LEU	Affected	Not Affected	More Stable	Affected	Affected	Not Affected
22	1	CTG→CGG	LEU→ARG	Not Affected	Not Affected	Less Stable	Not Affected	Not Affected	Not Affected
22	1	CTG→CCG	LEU→PRO	Not Affected	Not Affected	Less Stable	Affected	Affected	Not Affected
26	1	GAG→AAG	GLU→LYS	Not Affected	Not Affected	Less Stable	Affected	Affected	Not Affected
27	1	CTG→CCG	LEU→PRO	Not Affected	Not Affected	Less Stable	Affected	Affected	Not Affected
37	2	CTT→CCT	LEU→PRO	Not Affected	Not Affected	Less Stable	Affected	Affected	Not Affected
38	2	TCC→TTC	SER→PHE	Not Affected	Not Affected	Less Stable	Affected	Affected	Not Affected
38	2	TCC→CCC	SER→PRO	Not Affected	Not Affected	Less Stable	Affected	Affected	Not Affected
39	2	TTG→TGG	LEU→TRP	Not Affected	Not Affected	Less Stable	Not Affected	Not Affected	Not Affected
42	2	GGC→GTC	GLY→VAL	Not Affected	Not Affected	More Stable	Not Affected	Not Affected	Not Affected
42	2	GGC→GAC	GLY→ASP	Not Affected	Not Affected	Uncertain	Affected	Affected	Not Affected
42	2	GGC→AGC	GLY→SER	Not Affected	Not Affected	Less Stable	Affected	Affected	Not Affected
42	2	GGC→GCC	GLY→ALA	Not Affected	Not Affected	Uncertain	Not Affected	Not Affected	Not Affected
45	2	GAG→GTG	GLU→VAL	Not Affected	Not Affected	Less Stable	Affected	Affected	Not Affected
45	2	GAG→GAC	GLU→ASP	Not Affected	Not Affected	Less Stable	Affected	Affected	Not Affected
45	2	GAG→GGG	GLU→GLY	Not Affected	Affected	Less Stable	Affected	Affected	Not Affected
45	2	GAG→AAG	GLU→LYS	Not Affected	Not Affected	Less Stable	Affected	Affected	Not Affected
45	2	GAG→GCG	GLU→ALA	Not Affected	Not Affected	Less Stable	Affected	Affected	Not Affected
45	2	GAG→CAG	GLU→GLN	Not Affected	Not Affected	Less Stable	Affected	Affected	Not Affected
49	2	GCT→GTT	ALA→VAL	Not Affected	Not Affected	Less Stable	Not Affected	Not Affected	Not Affected
52	2	CGC→GGC	ARG→GLY	Not Affected	Not Affected	Less Stable	Affected	Affected	Not Affected
53	2	GTC→ATC	VAL→ILE	Not Affected	Not Affected	Uncertain	Not Affected	Not Affected	Not Affected
57	2	AAC→AAA	ASN→LYS	Not Affected	Not Affected	Less Stable	Not Affected	Not Affected	Not Affected
72	2	CCT→CAT	PRO→HIS	Not Affected	Not Affected	More Stable	Affected	Affected	Not Affected
86	2	ATC→TTC	ILE→PHE	Not Affected	Not Affected	Less Stable	Not Affected	Not Affected	Not Affected
89	2	CTC→CGC	LEU→ARG	Not Affected	Not Affected	Less Stable	Not Affected	Not Affected	Not Affected
98	2	CGA→CTA	ARG→LEU	Not Affected	Not Affected	More Stable	Not Affected	Not Affected	Not Affected
110	2	GGG→GAG	GLY→GLU	Not Affected	Not Affected	Less Stable	Not Affected	Not Affected	Not Affected

116	2 GAG→GGG	GLU→GLY	Not Affected	Not Affected	Less Stable	Not Affected	Not Affected	Not Affected
126	2 TGG→GGG	TRP→GLY	Not Affected	Not Affected	Less Stable	Affected	Affected	Not Affected
137	2 CGG→TGG	ARG→TRP	Not Affected	Not Affected	Uncertain	Not Affected	Not Affected	Not Affected
139	2 CAC→TAC	HIS→TYR	Not Affected	Not Affected	More Stable	Affected	Affected	Not Affected
139	2 CAC→GAC	HIS→ASP	Not Affected	Not Affected	Less Stable	Affected	Affected	Not Affected
139	2 CAC→CGC	HIS→ARG	Not Affected	Affected	Less Stable	Affected	Affected	Not Affected
139	2 CAC→AAC	HIS→ASN	Not Affected	Not Affected	Less Stable	Affected	Affected	Not Affected
139	2 CAC→CCC	HIS→PRO	Affected	Not Affected	Less Stable	Affected	Affected	Not Affected
141	2 CAG→CGG	GLN→ARG	Not Affected	Not Affected	Uncertain	Affected	Affected	Not Affected
144	2 TTC→TGC	PHE→CYS	Not Affected	Not Affected	Less Stable	Not Affected	Not Affected	Not Affected
144	2 TTC→GTC	PHE→VAL	Not Affected	Not Affected	Less Stable	Not Affected	Not Affected	Not Affected
145	2 AGC→AGG	SER→ARG	Not Affected	Not Affected	Uncertain	Affected	Affected	Not Affected
146	2 TTC→TCC	PHE→SER	Not Affected	Affected	Less Stable	Affected	Affected	Not Affected
147	2 ATC→TTC	ILE→PHE	Not Affected	Not Affected	Uncertain	Not Affected	Not Affected	Not Affected
148	2 ACA→CCA	THR→PRO	Affected	Not Affected	Less Stable	Affected	Affected	Not Affected
157	2 TTG→TGG	LEU→TRP	Not Affected	Not Affected	Less Stable	Not Affected	Not Affected	Not Affected
158	2 GAC→GTC	ASP→VAL	Not Affected	Not Affected	More Stable	Affected	Affected	Not Affected
158	2 GAC→TAC	ASP→TYR	Not Affected	Affected	Uncertain	Affected	Affected	Not Affected
158	2 GAC→GAG	ASP→GLU	Not Affected	Affected	Less Stable	Affected	Affected	Not Affected
159	2 AGC→ATC	SER→ILE	Not Affected	Not Affected	More Stable	Affected	Affected	Not Affected
160	2 TCC→TTC	SER→PHE	Not Affected	Not Affected	More Stable	Not Affected	Not Affected	Not Affected
161	2 GGT→GAT	GLY→ASP	Not Affected	Not Affected	Less Stable	Affected	Affected	Not Affected
161	2 GGT→GTT	GLY→VAL	Not Affected	Not Affected	More Stable	Affected	Affected	Not Affected
161	2 GGT→CGT	GLY→ARG	Not Affected	Not Affected	Uncertain	Affected	Affected	Not Affected
161	2 GGT→TGT	GLY→CYS	Not Affected	Not Affected	Less Stable	Affected	Affected	Not Affected
161	2 GGT→AGT	GLY→SER	Not Affected	Not Affected	Uncertain	Affected	Affected	Not Affected
163	2 GCC→GAC	ALA→ASP	Not Affected	Not Affected	Less Stable	Affected	Affected	Not Affected
164	2 TTT→TGT	PHE→CYS	Not Affected	Affected	Less Stable	Not Affected	Not Affected	Not Affected
165	2 GCT→ACT	ALA→THR	Not Affected	Not Affected	Less Stable	Not Affected	Not Affected	Not Affected
165	2 GCT→CCT	ALA→PRO	Not Affected	Not Affected	Less Stable	Affected	Affected	Not Affected
167	2 GTT→TTT	VAL→PHE	Not Affected	Not Affected	Less Stable	Not Affected	Not Affected	Not Affected
168	2 GGG→AGG	GLY→ARG	Not Affected	Not Affected	Less Stable	Affected	Affected	Not Affected
169	2 GCC→GAC	ALA→ASP	Not Affected	Not Affected	Less Stable	Affected	Affected	Not Affected

170	2 TGC→CGC	CYS→ARG	Not Affected	Not Affected	Less Stable	Affected	Affected	Not Affected
170	2 TGC→TAC	CYS→TYR	Not Affected	Not Affected	Less Stable	Affected	Affected	Not Affected
173	2 CTG→CCG	LEU→PRO	Affected	Not Affected	Less Stable	Affected	Affected	Not Affected
176	2 CGG→CAG	ARG→GLN	Not Affected	Not Affected	Less Stable	Not Affected	Not Affected	Not Affected
176	2 CGG→TGG	ARG→TRP	Not Affected	Not Affected	Uncertain	Not Affected	Not Affected	Not Affected
177	2 GAT→GTT	ASP→VAL	Not Affected	Not Affected	Uncertain	Affected	Affected	Not Affected
177	2 GAT→TAT	ASP→TYR	Not Affected	Affected	Uncertain	Affected	Affected	Not Affected
180	2 CTC→CGC	LEU→ARG	Not Affected	Not Affected	Less Stable	Not Affected	Not Affected	Not Affected
181	2 GCC→CCC	ALA→PRO	Not Affected	Not Affected	Less Stable	Affected	Affected	Not Affected
181	2 GCC→TCC	ALA→SER	Not Affected	Not Affected	Less Stable	Not Affected	Not Affected	Not Affected
182	2 CTG→CCG	LEU→PRO	Not Affected	Not Affected	Less Stable	Affected	Affected	Not Affected
184	2 GAG→AAG	GLU→LYS	Not Affected	Not Affected	Uncertain	Affected	Affected	Not Affected
184	2 GAG→CAG	GLU→GLN	Not Affected	Not Affected	Less Stable	Affected	Affected	Not Affected
185	2 GAT→GCT	ASP→ALA	Not Affected	Not Affected	Less Stable	Not Affected	Not Affected	Not Affected
186	3 CAT→GAT	HIS→ASP	Not Affected	Not Affected	Less Stable	Affected	Affected	Not Affected
186	3 CAT→CGT	HIS→ARG	Not Affected	Not Affected	Uncertain	Affected	Affected	Affected
188	3 TGG→TGC	TRP→CYS	Not Affected	Not Affected	Less Stable	Not Affected	Not Affected	Not Affected
188	3 TGG→CGG	TRP→ARG	Not Affected	Not Affected	Less Stable	Affected	Affected	Not Affected
188	3 TGG→TCG	TRP→SER	Not Affected	Not Affected	Less Stable	Not Affected	Not Affected	Not Affected
189	3 GTA→GAA	VAL→GLU	Not Affected	Not Affected	Less Stable	Not Affected	Not Affected	Not Affected
193	3 CCC→CTC	PRO→LEU	Not Affected	Not Affected	Uncertain	Not Affected	Not Affected	Not Affected
194	3 AAT→AGT	ASN→SER	Not Affected	Not Affected	More Stable	Not Affected	Not Affected	Not Affected
197	3 CAG→AAG	GLN→LYS	Not Affected	Not Affected	Uncertain	Affected	Affected	Not Affected
198	3 ACA→ATA	THR→ILE	Not Affected	Not Affected	More Stable	Affected	Affected	Not Affected
200	3 GAG→GGG	GLU→GLY	Not Affected	Affected	Less Stable	Affected	Affected	Not Affected
201	3 GTC→GGC	VAL→GLY	Not Affected	Not Affected	Less Stable	Not Affected	Not Affected	Not Affected
202	3 ACC→ATC	THR→ILE	Not Affected	Not Affected	Less Stable	Affected	Affected	Not Affected
204	3 CAC→GAC	HIS→ASP	Not Affected	Affected	Less Stable	Not Affected	Not Affected	Not Affected
220	3 GTG→ATG	VAL→MET	Not Affected	Affected	Uncertain	Not Affected	Not Affected	Not Affected
225	3 TGG→CGG	TRP→ARG	Not Affected	Not Affected	Less Stable	Affected	Affected	Not Affected
225	3 TGG→TGC	TRP→SER	Not Affected	Affected	Less Stable	Affected	Affected	Not Affected
225	3 TGG→TTG	TRP→LEU	Not Affected	Not Affected	Less Stable	Affected	Affected	Not Affected
228	3 CTG→CCG	LEU→PRO	Not Affected	Not Affected	Less Stable	Affected	Affected	Not Affected

230	3 GGA→AGA	GLY→ARG	Not Affected	Affected	Uncertain	Not Affected	Not Affected	Not Affected
231	3 TCA→CCA	SER→PRO	Not Affected	Not Affected	Less Stable	Affected	Affected	Not Affected
234	3 CGC→CAC	ARG→HIS	Not Affected	Not Affected	Less Stable	Affected	Affected	Not Affected
234	3 CGC→CTC	ARG→LEU	Not Affected	Not Affected	Less Stable	Affected	Affected	Not Affected
236	3 GAC→CAC	ASP→HIS	Not Affected	Not Affected	Less Stable	Affected	Affected	Not Affected
240	3 GAG→AAG	GLU→LYS	Not Affected	Not Affected	Less Stable	Affected	Affected	Not Affected
246	3 TGT→TAT	CYS→TYR	Not Affected	Not Affected	Less Stable	Not Affected	Not Affected	Not Affected
246	3 TGT→TTT	CYS→PHE	Not Affected	Not Affected	Less Stable	Not Affected	Not Affected	Not Affected
246	3 TGT→CGT	CYS→ARG	Not Affected	Not Affected	Less Stable	Affected	Affected	Not Affected
247	3 GCC→GTC	ALA→VAL	Not Affected	Not Affected	Less Stable	Not Affected	Not Affected	Not Affected
252	3 ATT→ACT	ILE→THR	Not Affected	Affected	Less Stable	Not Affected	Not Affected	Not Affected
254	3 CTG→CCG	LEU→PRO	Not Affected	Not Affected	Uncertain	Affected	Affected	Not Affected
258	4 TCG→TTG	SER→LEU	Not Affected	Affected	Uncertain	Affected	Affected	Not Affected
258	4 TCG→TGG	SER→TRP	Not Affected	Affected	Uncertain	Affected	Affected	Not Affected
258	4 TCG→CCG	SER→PRO	Not Affected	Not Affected	Less Stable	Affected	Affected	Not Affected
260	4 GAG→AAG	GLU→LYS	Not Affected	Not Affected	Uncertain	Not Affected	Not Affected	Not Affected
261	4 CTT→TTT	LEU→PHE	Not Affected	Not Affected	Less Stable	Not Affected	Not Affected	Not Affected
263	4 CAG→CAC	GLN→HIS	Not Affected	Affected	Uncertain	Affected	Affected	Not Affected
264	4 CTG→CGG	LEU→ARG	Not Affected	Not Affected	Less Stable	Not Affected	Not Affected	Not Affected
264	4 CTG→CCG	LEU→PRO	Not Affected	Not Affected	Less Stable	Affected	Affected	Not Affected
265	4 CAG→CGG	GLN→ARG	Not Affected	Not Affected	Less Stable	Affected	Affected	Not Affected
265	4 CAG→CCG	GLN→PRO	Not Affected	Not Affected	Less Stable	Affected	Affected	Not Affected
269	4 CTC→CCC	LEU→PRO	Not Affected	Not Affected	Less Stable	Affected	Affected	Not Affected
272	4 CTC→CCC	LEU→PRO	Not Affected	Not Affected	Less Stable	Affected	Affected	Not Affected
278	4 CTG→CCG	LEU→PRO	Not Affected	Not Affected	Less Stable	Not Affected	Not Affected	Not Affected
279	4 GAA→GCA	GLU→ALA	Not Affected	Not Affected	Less Stable	Affected	Affected	Not Affected
280	4 AGG→AAG	ARG→LYS	Not Affected	Affected	Less Stable	Not Affected	Not Affected	Not Affected
282	4 CCC→CAC	PRO→HIS	Not Affected	Not Affected	Less Stable	Not Affected	Not Affected	Not Affected
282	4 CCC→CTC	PRO→LEU	Not Affected	Not Affected	More Stable	Not Affected	Not Affected	Not Affected
286	4 GGG→AGG	GLY→ARG	Not Affected	Not Affected	Uncertain	Not Affected	Not Affected	Not Affected
289	4 GCA→CCA	ALA→PRO	Not Affected	Not Affected	Less Stable	Affected	Affected	Not Affected
289	4 GCA→GTA	ALA→VAL	Not Affected	Not Affected	Less Stable	Not Affected	Not Affected	Not Affected
289	4 GCA→GAA	ALA→GLU	Not Affected	Not Affected	Less Stable	Not Affected	Not Affected	Not Affected

289	4	GCA→CAA	ALA→GLN	Not Affected	Not Affected	Less Stable	Not Affected	Not Affected	Not Affected
291	4	CTA→CCA	LEU→PRO	Not Affected	Not Affected	Less Stable	Affected	Affected	Not Affected
294	4	CTG→CCG	LEU→PRO	Affected	Not Affected	Less Stable	Affected	Affected	Not Affected
310	5	GGC→CGC	GLY→ARG	Not Affected	Not Affected	Uncertain	Not Affected	Not Affected	Not Affected
310	5	GGC→GAC	GLY→ASP	Not Affected	Not Affected	Uncertain	Not Affected	Not Affected	Not Affected
314	6	GCC→CCC	ALA→PRO	Not Affected	Not Affected	Less Stable	Affected	Affected	Not Affected
316	6	ACC→CCC	THR→PRO	Not Affected	Not Affected	Uncertain	Affected	Affected	Not Affected
319	6	CGG→CCG	ARG→PRO	Not Affected	Not Affected	Less Stable	Not Affected	Not Affected	Not Affected
320	6	GAT→TAT	ASP→TYR	Not Affected	Affected	More Stable	Affected	Affected	Not Affected
322	6	CAC→CGC	HIS→ARG	Not Affected	Not Affected	Less Stable	Affected	Affected	Not Affected
322	6	CAC→TAC	HIS→TYR	Not Affected	Not Affected	Less Stable	Affected	Affected	Not Affected
325	6	CCC→CGC	PRO→ARG	Not Affected	Not Affected	Uncertain	Not Affected	Not Affected	Not Affected
325	6	CCC→CTC	PRO→LEU	Not Affected	Not Affected	More Stable	Not Affected	Not Affected	Not Affected
330	6	GCT→CCT	ALA→PRO	Not Affected	Not Affected	Less Stable	Affected	Affected	Not Affected
335	6	CGC→CCC	ARG→PRO	Not Affected	Not Affected	Less Stable	Affected	Affected	Affected
342	7	GCC→GAC	ALA→ASP	Not Affected	Not Affected	Less Stable	Not Affected	Not Affected	Not Affected
342	7	GCC→CCC	ALA→PRO	Not Affected	Not Affected	Less Stable	Affected	Affected	Not Affected
343	7	CTG→CCG	LEU→PRO	Not Affected	Not Affected	Less Stable	Affected	Affected	Not Affected
345	7	GCC→TCC	ALA→THR	Not Affected	Not Affected	Less Stable	Affected	Affected	Not Affected
347	7	GCG→CCG	ALA→PRO	Not Affected	Not Affected	Less Stable	Affected	Affected	Not Affected
349	7	ACG→AGG	THR→ARG	Not Affected	Not Affected	Less Stable	Affected	Affected	Not Affected
353	7	ATC→AAC	ILE→ASN	Not Affected	Not Affected	Less Stable	Not Affected	Not Affected	Not Affected
355	7	GAC→GTC	ASP→VAL	Not Affected	Not Affected	Uncertain	Affected	Affected	Not Affected
356	7	TAC→AAC	TYR→ASN	Not Affected	Not Affected	Less Stable	Affected	Affected	Not Affected
358	7	TAC→GAC	TYR→ASP	Not Affected	Not Affected	Less Stable	Not Affected	Not Affected	Not Affected
359	7	TGC→TTC	CYS→PHE	Not Affected	Not Affected	Less Stable	Affected	Affected	Not Affected
360	7	CGG→TGG	ARG→TRP	Not Affected	Not Affected	Uncertain	Not Affected	Not Affected	Not Affected
362	7	GAC→CAC	ASP→HIS	Not Affected	Not Affected	Less Stable	Affected	Affected	Not Affected
364	7	GAG→AAG	GLU→LYS	Not Affected	Not Affected	Uncertain	Affected	Affected	Not Affected
369	7	TTC→TGC	PHE→CYS	Not Affected	Not Affected	Less Stable	Affected	Affected	Not Affected
371	7	GAA→GAT	GLU→ASP	Not Affected	Not Affected	Less Stable	Not Affected	Not Affected	Not Affected
373	7	GCC→GAC	ALA→ASP	Not Affected	Not Affected	Less Stable	Not Affected	Not Affected	Not Affected
373	7	GCC→CCC	ALA→PRO	Not Affected	Not Affected	Less Stable	Affected	Affected	Not Affected

377	7	ATC→ATG	ILE→MET	Not Affected	Not Affected	Less Stable	Not Affected	Not Affected	Not Affected
378	7	CCC→GCC	PRO→ALA	Not Affected	Affected	Less Stable	Not Affected	Not Affected	Not Affected
378	7	CCC→CTC	PRO→LEU	Not Affected	Affected	More Stable	Not Affected	Not Affected	Not Affected
378	7	CCC→TCC	PRO→SER	Not Affected	Affected	Less Stable	Affected	Affected	Not Affected
380	7	CTG→CCG	LEU→PRO	Not Affected	Affected	Less Stable	Affected	Affected	Not Affected
381	7	CTG→CCG	LEU→PRO	Not Affected	Not Affected	Less Stable	Affected	Affected	Not Affected
390	8	GCG→GTG	ALA→VAL	Not Affected	Not Affected	Uncertain	Not Affected	Not Affected	Not Affected
395	8	CCG→CGG	PRO→ARG	Not Affected	Not Affected	More Stable	Not Affected	Not Affected	Not Affected
415	8	TTC→TTA	PHE→LEU	Not Affected	Not Affected	Less Stable	Not Affected	Not Affected	Not Affected
416	8	GCC→CCC	ALA→PRO	Not Affected	Not Affected	Less Stable	Affected	Affected	Not Affected
418	8	CTG→CGG	LEU→ARG	Not Affected	Not Affected	Less Stable	Not Affected	Not Affected	Not Affected
418	8	CTG→CCG	LEU→PRO	Not Affected	Not Affected	Less Stable	Affected	Affected	Not Affected
419	8	CTG→CCG	LEU→PRO	Not Affected	Not Affected	Less Stable	Affected	Affected	Not Affected
419	8	CTG→CAG	LEU→GLN	Not Affected	Not Affected	Less Stable	Not Affected	Not Affected	Not Affected
420	8	CGA→CCA	ARG→PRO	Not Affected	Not Affected	Less Stable	Affected	Affected	Not Affected
423	8	GAC→AAC	ASP→ASN	Not Affected	Not Affected	Less Stable	Affected	Affected	Not Affected
423	8	GAC→CAC	ASP→HIS	Not Affected	Not Affected	Less Stable	Affected	Affected	Not Affected
424	8	GGC→GTC	GLY→VAL	Not Affected	Not Affected	More Stable	Not Affected	Not Affected	Not Affected
425	8	ATC→AAC	ILE→ASN	Not Affected	Not Affected	Less Stable	Not Affected	Not Affected	Not Affected
426	8	TGC→TAC	CYS→TYR	Not Affected	Affected	Less Stable	Affected	Affected	Not Affected
428	8	TGG→CGG	TRP→ARG	Not Affected	Not Affected	Less Stable	Not Affected	Not Affected	Not Affected
428	8	TGG→TCG	TRP→SER	Not Affected	Not Affected	Less Stable	Affected	Affected	Not Affected
432	8	AGT→AGA	SER→ARG	Not Affected	Not Affected	Less Stable	Affected	Affected	Not Affected
432	8	AGT→ATT	SER→ILE	Not Affected	Not Affected	Less Stable	Affected	Affected	Not Affected
434	9	ACG→AAG	THR→LYS	Not Affected	Not Affected	Uncertain	Not Affected	Not Affected	Not Affected
441	9	TGG→TGT	TRP→CYS	Not Affected	Not Affected	Less Stable	Affected	Affected	Not Affected
441	9	TGG→TCG	TRP→SER	Not Affected	Not Affected	Less Stable	Affected	Affected	Not Affected
448	9	TCC→TAC	SER→TYR	Not Affected	Not Affected	Uncertain	Affected	Affected	Not Affected
449	9	CTA→CCA	LEU→PRO	Not Affected	Not Affected	Less Stable	Affected	Affected	Not Affected
452	9	TTT→TCT	PHE→SER	Not Affected	Not Affected	Less Stable	Affected	Affected	Not Affected
452	9	TTT→CTT	PHE→LEU	Not Affected	Not Affected	Less Stable	Affected	Affected	Not Affected
457	9	CGG→TGG	ARG→TRP	Not Affected	Not Affected	Uncertain	Affected	Affected	Not Affected
476	9	TGG→TGT	TRP→CYS	Not Affected	Not Affected	Uncertain	Affected	Affected	Not Affected

507	10 AAG→ATG	LYS→MET	Not Affected	Not Affected	Uncertain	Not Affected	Not Affected	Not Affected
508	10 GGC→GAC	GLY→ASP	Not Affected	Not Affected	Uncertain	Not Affected	Not Affected	Not Affected
513	10 CAG→AAG	GLN→LYS	Not Affected	Not Affected	Uncertain	Not Affected	Not Affected	Not Affected
521	10 CGG→GGG	ARG→GLY	Not Affected	Not Affected	Less Stable	Not Affected	Not Affected	Not Affected
537	10 GGC→TGC	GLY→CYS	Not Affected	Not Affected	Uncertain	Not Affected	Not Affected	Not Affected
540	10 GCT→GTT	ALA→VAL	Not Affected	Not Affected	Uncertain	Not Affected	Not Affected	Not Affected
545	10 CCC→TCC	PRO→SER	Not Affected	Not Affected	Less Stable	Not Affected	Not Affected	Not Affected
546	10 ACA→GCA	THR→ALA	Not Affected	Not Affected	Uncertain	Affected	Affected	Not Affected
548	10 TCA→TTA	SER→LEU	Not Affected	Not Affected	More Stable	Not Affected	Not Affected	Not Affected
549	10 CCA→TCA	PRO→SER	Not Affected	Not Affected	Less Stable	Affected	Affected	Not Affected
555	10 GTG→CTG	VAL→LEU	Not Affected	Not Affected	Uncertain	Not Affected	Not Affected	Not Affected
557	10 ACT→TCT	THR→SER	Not Affected	Not Affected	Less Stable	Affected	Affected	Not Affected
560	10 AGT→AAT	SER→ASN	Not Affected	Not Affected	Less Stable	Affected	Affected	Not Affected
562	10 AAG→GAG	LYS→GLU	Not Affected	Not Affected	Less Stable	Affected	Affected	Affected
563	10 ATG→AAG	MET→LYS	Not Affected	Not Affected	Less Stable	Affected	Affected	Not Affected
566	10 ATG→AGG	MET→ARG	Not Affected	Affected	Less Stable	Affected	Affected	Not Affected
566	10 ATG→ACG	MET→THR	Not Affected	Not Affected	Less Stable	Affected	Affected	Not Affected
566	10 ATG→AAG	MET→LYS	Not Affected	Not Affected	Less Stable	Affected	Affected	Not Affected
580	10 ATC→AAC	ILE→ASN	Not Affected	Not Affected	Less Stable	Not Affected	Not Affected	Not Affected
584	10 CTC→CCC	LEU→PRO	Not Affected	Not Affected	Less Stable	Affected	Affected	Not Affected
588	10 TCG→CCG	SER→PRO	Not Affected	Not Affected	Less Stable	Not Affected	Not Affected	Not Affected
